# Biogenesis and delivery of extracellular vesicles: harnessing the power of EVs for diagnostics and therapeutics

**DOI:** 10.3389/fmolb.2023.1330400

**Published:** 2024-01-03

**Authors:** Jivin Yu, Saba Sane, Ji-Eun Kim, Sehee Yun, Hyeon-Jai Kim, Kyeong Beom Jo, Jacob P. Wright, Nooshin Khoshdoozmasouleh, Kunwoo Lee, Ho Taek Oh, Keaton Thiel, Afrin Parvin, Xavier Williams, Claire Hannon, Hunsang Lee, Dae-Kyum Kim

**Affiliations:** ^1^ Department of Life Sciences, College of Life Sciences and Biotechnology, Korea University, Seoul, Republic of Korea; ^2^ Department of Cancer Genetics and Genomics, Roswell Park Comprehensive Cancer Center, Buffalo, NY, United States; ^3^ Department of Experimental Animal Research, Biomedical Research Institute, Seoul National University Hospital, Seoul, Republic of Korea; ^4^ College of Arts and Sciences, University at Buffalo, Buffalo, NY, United States; ^5^ Applied Technology Laboratory for Advanced Surgery (ATLAS) Studios Resource, Roswell Park Comprehensive Cancer Center, Buffalo, NY, United States

**Keywords:** extracellular vesicles, exosomes, ectosomes, biogenesis, delivery, uptake, network analysis

## Abstract

Extracellular vesicles (EVs) are membrane-enclosed particles secreted by a variety of cell types. These vesicles encapsulate a diverse range of molecules, including proteins, nucleic acids, lipids, metabolites, and even organelles derived from their parental cells. While EVs have emerged as crucial mediators of intercellular communication, they also hold immense potential as both biomarkers and therapeutic agents for numerous diseases. A thorough understanding of EV biogenesis is crucial for the development of EV-based diagnostic developments since the composition of EVs can reflect the health and disease status of the donor cell. Moreover, when EVs are taken up by target cells, they can exert profound effects on gene expression, signaling pathways, and cellular behavior, which makes these biomolecules enticing targets for therapeutic interventions. Yet, despite decades of research, the intricate processes underlying EV biogenesis by donor cells and subsequent uptake by recipient cells remain poorly understood. In this review, we aim to summarize current insights and advancements in the biogenesis and uptake mechanisms of EVs. By shedding light on the fundamental mechanisms governing EV biogenesis and delivery, this review underscores the potential of basic mechanistic research to pave the way for developing novel diagnostic strategies and therapeutic applications.

## 1 Introduction

Extracellular vesicles (EVs), which are lipid bilayer-enclosed, nano-sized vesicles, are ubiquitously secreted from various cell types. These mediate cell-to-cell communications and thus ensure life-sustaining physiological functions through the transport of diverse biological compounds (Li, 2021) while the lipid bilayer shields the cargo from harsh extracellular environments, such as digestive enzymes (Skotland et al., 2020). The use of EVs as biomarkers and therapeutic agents for numerous diseases is a highly active area of research. Several reviews on EV cell biology, lipid components, and clinical application with current research advances have been published recently (van Niel et al., 2018; Zhi et al., 2022; Ghadami and Dellinger, 2023).

Upon secretion, EVs mediate both paracrine and endocrine signaling by delivering their cargo to target cells which consequently regulates the behavior of recipient cells. Generally, EVs are enriched in cytoskeleton, cytoplasmic, heat shock, cell membrane, and vesicle transport proteins with a paucity of organellar proteins in the cell (Li, 2021; Kalluri and McAndrews, 2023). EVs are notably variable as compositions can differ considerably based on the physiological state of their originating cells (Li, 2021). Depending on the context, certain EVs may be particularly enriched with endosome-associated proteins (e.g., Rab GTPase, SNAP receptor proteins called SNAREs, annexins, and flotillins) involved in the biogenesis of multivesicular bodies (MVB). Additionally, there can be variations in the types and levels of nucleic acids in EV; ranging from complete messenger RNA (mRNA), fragments of mRNA, long non-coding RNA (lncRNA), micro RNA (miRNA), ribosomal RNA (rRNA), single-stranded DNA (ssDNA), and mitochondrial DNA (mtDNA) to double-stranded DNA (dsDNA). Another variable is the EV membrane composition carrying ligands and receptors from source cells which are crucial determinants in the EV uptake process on recipient cells as compositional changes in the membrane can alter target specificity (Zhang X. et al., 2021).

Notably, EVs have gained attention for their role in pathophysiological processes (Busatto et al., 2021; Sun et al., 2022) like cell death, inflammation (Sanwlani and Gangoda, 2021), angiogenesis (Guo et al., 2017), and even cancer progression. With the following evidence, cancer-derived EVs exhibit distinct lipid and protein compositions when compared with normal cells. Strikingly, malignancy can be a result of EVs transferring their oncogenic materials to normal cells (Tai et al., 2019). EVs can further facilitate tumor invasion and metastasis by delivering matrix metalloproteinases (MMPs) and other proteases to the target sites (Han et al., 2019) and stimulating angiogenesis by the transfer of pro-angiogenic factors to endothelial cells (Phelps et al., 2023). Collectively, these findings suggest that EVs are key players in mediating communications between cancer cells and their surrounding stroma, where they contribute to shaping the tumor microenvironment (Kim et al., 2002; Berumen Sánchez et al., 2021).

Since EVs can be isolated from assorted biological fluids such as blood, urine, and saliva and components and functions of EVs are distinct for each EV source, their detection in non-invasive liquid biopsies shows promise for diagnostic marker applications (Cheng et al., 2014; Chiabotto et al., 2019). Therapeutically, EVs are also being investigated as novel nanoparticle drug carriers, necessitating an in-depth understanding of cargo selection during EV biogenesis and target cell specificity during uptake. For instance, EVs produced by mesenchymal stem cells have immunomodulatory properties and are currently being trialed for treating inflammatory diseases (Lai et al., 2010; Sun et al., 2018). Moreover, EVs can be engineered to carry specific therapeutic molecules to specific cells and tissues (Aqil et al., 2019; Vakhshiteh et al., 2021).

In this review, we first provide an overview of current understanding of EV biogenesis, delivery pathways, and respective cargos. On another note, we delve into the biogenesis of diverse EV types, discuss their uptake mechanisms, and examine the functional roles of EV regulators using public EV databases (e.g., EVpedia) to assist with assessments of their potential both as disease biomarkers and therapeutic agents.

## 2 EV biogenesis

**FIGURE 1:**
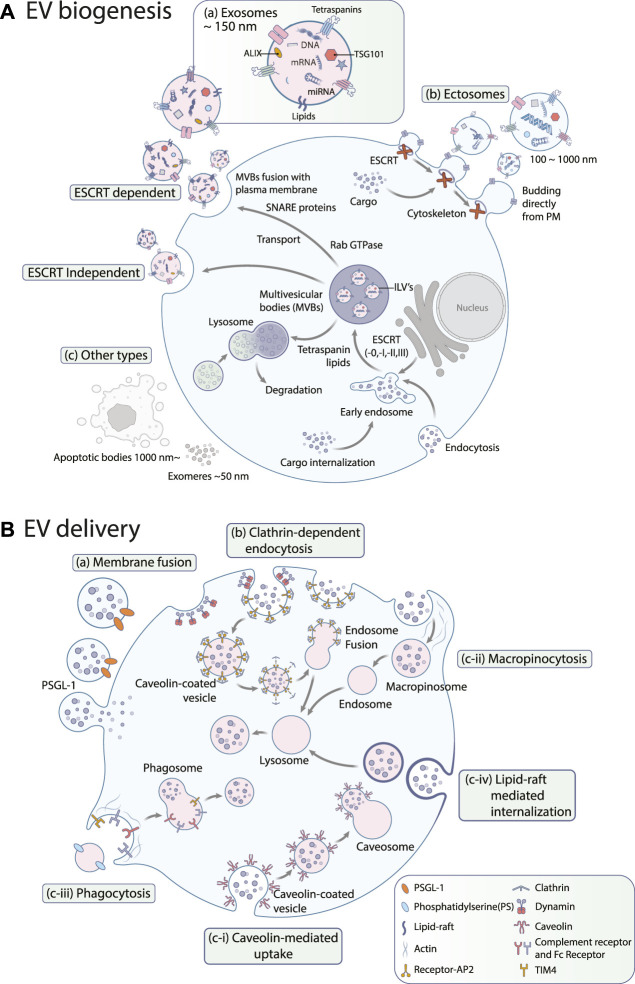
**(A)** EV biogenesis. EVs are largely categorized into **(a)** exosomes, **(b)** ectosomes, and **(c)** other types such as apoptotic bodies or exomeres. Exosome biogenesis occurs through both **(a-i)** ESCRT-dependent and **(a-ii)** ESCRT-independent pathways. PM: plasma membrane **(B)** EV uptake pathways. Exosomes can be internalized by **(a)** membrane fusion, **(b)** clathrin-dependent endocytosis, and **(c)** clathrin-independent endocytosis including **(c-i)** caveolin-mediated uptake, **(c-ii)** macropinocytosis, **(c-iii)** phagocytosis, and **(c-iv)** lipid raft-mediated internalization. PSGL-1: P-selectin glycoprotein ligand-1, PS: phosphatidylserine, AP2: adipocyte protein 2, TIM4: T-cell immunoglobulin and mucin domain containing 4.

EVs are broadly classified into three categories (Figure 1A) based on their biogenesis pathway: exosomes, ectosomes, and apoptotic bodies (Busatto et al., 2021). Exosomes are intraluminal vesicles, typically less than 150 nm in diameter, originating from the fusion of MVBs with the plasma membrane. Contrarily, ectosomes, or microvesicles, ranging from approximately 100–1,000 nm, are produced by budding and shedding from the plasma membrane (Li, 2021; Lim et al., 2021; Han et al., 2022). Similarly, exosomes and ectosomes mediate intercellular communications. The tetraspanin protein family has been identified as an essential cellular effector during the biogenesis of these vesicles, though their specific functions differ between exosome and ectosome formation (Perez-Hernandez et al., 2013). Apoptotic bodies, on the other hand, are released during programmed cell death, i.e., apoptosis, in the form of cell vesicles (Nagata, 2018).

### 2.1 Exosomes

Exosomes originate from the inward budding of the endosomal membrane, which leads to the formation of MVBs (Zhang et al., 2019), as shown in Figure 1A. These MVBs either fuse with lysosomes for degradation or merge with the plasma membrane to release exosomes (Han et al., 2022). The biogenesis of exosomes relies on the coordination of multiple pathways and can be classified into two primary routes: the endosomal sorting complex required for transport (ESCRT)-dependent and ESCRT-independent pathways. The ESCRT-dependent pathway involves the formation of intraluminal vesicles (ILVs) by the sequential action of ESCRT-0, -I, -II, and -III complexes, including ALIX, TSG101, and VPS4 (Baietti et al., 2012). On the contrary, the ESCRT-independent pathway involves formation of ILVs by tetraspanin-enriched microdomains (TEMs) and lipid rafts. Tetraspanins, including CD9, CD81, and CD63, are abundant in TEMs and regulate cargo sorting and packaging into exosomes (van Niel et al., 2011). Recent models of exosome release have revealed a new function for the endoplasmic reticulum. At the contact sites between the endoplasmic reticulum and late endosome membranes, it plays a crucial role in controlling the movement and maturation of late endosomes, which is associated with the activity of small GTPases. These processes are vital for the fusion of MVBs with the plasma membrane, leading to the release of exosomes (Gurung et al., 2021). Small Rab GTPases (RAB27a/b, RAB11, RAB7, RAB35) govern vesicle budding and motility, thereby facilitating the transport of MVBs for exosome release. Additional players include members of SNARE proteins (Vamp7, YKT6), which are indispensable for the fusion of MVBs with the plasma membrane (Xie et al., 2022), syndecan heparan sulfate proteoglycans, phospholipase D2 (PLD2), ADP ribosylation factor 6 (ARF6), and syntenin (Ghossoub et al., 2014).

### 2.2 Ectosomes

Ectosomes are another type of extracellular vesicle formed by the outward budding of the plasma membrane, as shown in Figure 1A. Unlike exosomes, ectosome biogenesis does not rely heavily on the endosomal trafficking pathways (Lim et al., 2021). The factors involved in ectosome biogenesis remain elusive; however, formation of ectosomes on the plasma membrane requires rigorous rearrangements of membrane and cytoskeletal constituents (Kalra et al., 2016; Meldolesi, 2021). During nucleation, proteins with lipid anchor modifications (e.g., myristoylated, palmitoylated) accumulate in the lumen to initiate membrane curvature formation for budding (Cocucci and Meldolesi, 2015). Transmembrane proteins and lipids also cluster in specific membrane domains, and ESCRT-I subunits are recruited to the plasma membrane, respectively (Nabhan et al., 2012). A small GTPase, ARF, is also known to regulate cargo assembly and secretion of ectosomes (Sedgwick et al., 2015). In the final budding process, ectosomes must be pinched off from the plasma membrane. For this process, loosening the cytoskeleton coincides with sorting cytosolic proteins and RNA molecules into ectosomes (Cocucci and Meldolesi, 2015). Ca^2+^ induces membrane reorganization and cytoskeleton disassembly, as well as ESCRT-III complexes are required for the pitching-off and release of ectosomes (Gurunathan et al., 2021; Sun et al., 2021).

### 2.3 Other types: apoptotic bodies, exomers, etc.

Other types of EVs include apoptotic bodies and nonvesicular nanoparticles such as exomeres, which deserve attention here as well (Figures 1A–C). Apoptotic bodies emerge from cells during the final stage of apoptosis and were previously regarded as mere debris of dead cells (Yu et al., 2023). They range from 50 nm to 3 μm in size and contain DNA fragments, histones, and/or immature glyco-epitopes. Like other EV types, their composition reflects their cellular origins. Upon uptake by other cells, apoptotic bodies can trigger anti-inflammatory or tolerogenic responses (Mohan et al., 2020). This has led to some controversy. While some researchers support the classification of apoptotic bodies as a subclass of EV based on their similarities in size and contents, others view apoptotic bodies solely as dead cell remnants (Caruso and Poon, 2018). The precise mechanisms and genes involved in forming apoptotic bodies are not yet fully understood. However, emerging evidence suggests that apoptotic bodies are formed through a process called apoptotic cell disassembly, characterized by a series of tightly regulated morphological steps (Santavanond et al., 2021).

Recently, exomeres and supermeres, generally smaller than 50 nm in diameter, have been identified as additional nonvesicular nanoparticles. Exomeres exhibit distinct proteomic profiles and bio-distribution patterns compared to small EVs. Similarly, supermeres are enriched with RNAs and show enhanced accumulation in tissues compared with exomeres and small EVs (Zhang Q. et al., 2021).

## 3 EV delivery

EVs facilitate the exchange of information between cells. Upon uptake via various mechanisms (Figure 1B), EVs can alter the biological characteristics and functions of recipient cells. These cellular effectors can trigger intracellular signaling pathways in recipient cells through surface ligand–receptor interactions, thereby delivering their contents into these cells. Changes in gene expression and disrupted signaling activities via EV uptake can lead to phenotypic modifications, instigating disease onset and progression (Kwok et al., 2021). This underscores the potential for EVs in novel therapeutic avenues.

The mechanism of EV uptake varies depending on the specific type of EV and the recipient cell. Broadly speaking, uptake can be categorized into three primary mechanisms: membrane fusion, clathrin-dependent endocytosis, and clathrin-independent endocytosis which includes both pinocytosis and phagocytosis. Furthermore, lipid rafts and specific protein–protein interactions have been identified as factors in the EV uptake process (Mulcahy et al., 2014).

### 3.1 Cell membrane fusion

The most straightforward mechanism of EV uptake is through cell surface membrane fusion (Figures 1B-a), which involves direct fusion of the EV membrane with the plasma membrane of the target cell. This mechanism is reminiscent of how some viruses infect cells by fusing their viral envelopes with the cell membrane (Conner and Schmid, 2003). However, any alterations in pH, temperature, lipid composition, and membrane can impact EV fusion (Perissinotto et al., 2021). This mechanism allows luminal and membrane-associated cargo transfer without involving endocytic or lysosomal pathways (Morandi et al., 2022). Fusion process can be inhibited by either annexin V or a PSGL-1 antibody, highlighting the crucial role of these proteins (Del Conde et al., 2005). Nevertheless, the molecular mechanisms and regulatory factors mediating this process are still poorly understood and warrant further investigation.

### 3.2 Clathrin-dependent endocytosis

Clathrin-dependent endocytosis (Figure 1B-b) is one of the main routes for EV uptake (Mulcahy et al., 2014). Various cell types adopt this uptake route, including macrophages (Feng et al., 2010), ovarian cancer cells (Escrevente et al., 2011), colon cancer cells (Horibe et al., 2018), hepatocellular carcinoma cells (Xu et al., 2023), and neuronal cells (Milosevic, 2018). The pathway requires plasma membrane to form clathrin-coated pits, and the EVs are subsequently taken up into the cell in clathrin-coated vesicles following the scission process. This step is catalyzed by the large GTPase dynamin, cutting the neck of the invaginated membrane (Kaksonen and Roux, 2018). Several studies have shown that inhibiting dynamin-2 reduces EV uptake by recipient cells (Barrès et al., 2010; Feng et al., 2010). After internalization, the clathrin coat is removed, which allows the EVs to fuse with endosomes (Mettlen et al., 2018).

### 3.3 Caveolin-mediated uptake

Caveolin-mediated uptake (Figure 1B-c-i) involves the formation of small invaginations on the plasma membrane called caveolae, which can be formed by the expression of a small integral membrane protein called caveolin. Each caveola has around 140–150 caveolin-1 (Kiss and Botos, 2009). Caveolin-1 forms dimers, which recruit cavin proteins (CAVIN1/2/3/4) to the caveolae. It also binds cholesterols and brings them onto the cellular surface for uptake and intracellular trafficking. Caveolar vesicles are highly organized and enriched in saturated phospholipids, sphingolipids, plasmenylethalomines, and cholesterol (Haddad et al., 2020).

### 3.4 Macropinocytosis

Macropinocytosis (Figure 1B-c-ii) is an endocytic pathway that involves the formation of large, actin-driven membrane protrusions called macropinosomes. These protrusions extend from the cell surface and engulf extracellular material, forming large vesicles within the cell. The macropinosomes then fuse with endosomes, which allows the internalized material to be processed and utilized by the cell. This process is utilized by specific cell types, such as macrophages and microglia, which play important roles in immune responses and clearance of cellular debris. The process of macropinocytosis is dependent on the actin cytoskeleton, phosphatidylinositol 3-kinase activity, and dynamin-2 function (Petrovčíková et al., 2018).

### 3.5 Phagocytosis

Phagocytosis (Figure 1B-c-iii) is a process that facilitates the internalization of large particles by cells through receptor-mediated mechanisms without requiring direct contact with internalized molecules or the extension of membrane ruffles, and the internalization of EVs is one of its application (Gordon, 2016). Phagocytic cells internalize EVs more efficiently through this process (Feng et al., 2010). This efficiency is dependent on the phagocytes’ differentiation states (Czernek et al., 2015). Phagocytosis of EVs is facilitated by various factors, including phosphatidylserine (PS), which is enriched on the outer membrane of EVs and implicated to play a role in facilitating EV entry into recipient cells (Fomina et al., 2003). Phosphoinositide 3-kinases (PI3Ks) are another key regulator that enables membrane insertion into forming phagosomes. PI3K inhibitors, namely, wortmannin and LY294002, can be used to inhibit EV uptake in a dose-dependent manner (Feng et al., 2010). Similarly, pharmacological inhibition of ERK1/2 inhibited EV uptake, suggesting that these signaling pathways may also contribute to EV uptake (Christianson et al., 2013).

### 3.6 Lipid raft-mediated internalization

Lipid rafts are detergent-resistant membrane microdomains (Mulcahy et al., 2014) enriched in cholesterol, glycosphingolipids, and glycosyl-phosphatidylinositol (GPI)-anchored proteins. These rafts act as organizing centers for signaling molecules (Nabi and Le, 2003) and internalization of EVs (Figure 1B-c-iv), which was proved by reduced EV uptake via inhibitors affecting lipid rafts (Izquierdo-Useros et al., 2009). Imaging studies revealed that poor co-localization of fluorescently labeled EVs was observed with caveolin-1 (Koumangoye et al., 2011; Svensson et al., 2013). This suggests that the lipid rafts used by EVs are caveolae-independent (Escrevente et al., 2011; Svensson et al., 2013). These lipid rafts can also be found in planar regions of the plasma membrane marked by a family of proteins called flotillins (Palecek et al., 1996). Flotillins positively regulate lipid raft-mediated endocytosis (Phuyal et al., 2014), which is independent of clathrin and caveolin (Glebov et al., 2006; Otto and Nichols, 2011; Meister and Tikkanen, 2014). The role of lipid rafts in EV uptake was confirmed in several independent studies employing inhibitors of cholesterol and glycosphingolipid synthesis (Wang et al., 1991; Platt et al., 1994). For example, methyl-β-cyclodextrin reduces exosome uptake in breast cancer cells (Koumangoye et al., 2011). Overall, these findings support the hypothesis that lipid rafts are involved in the EV uptake mechanism; however, the route’s scale and precise mechanisms remain to be elucidated.

## 4 Network analysis of EV biogenesis and uptake regulators

**FIGURE 2:**
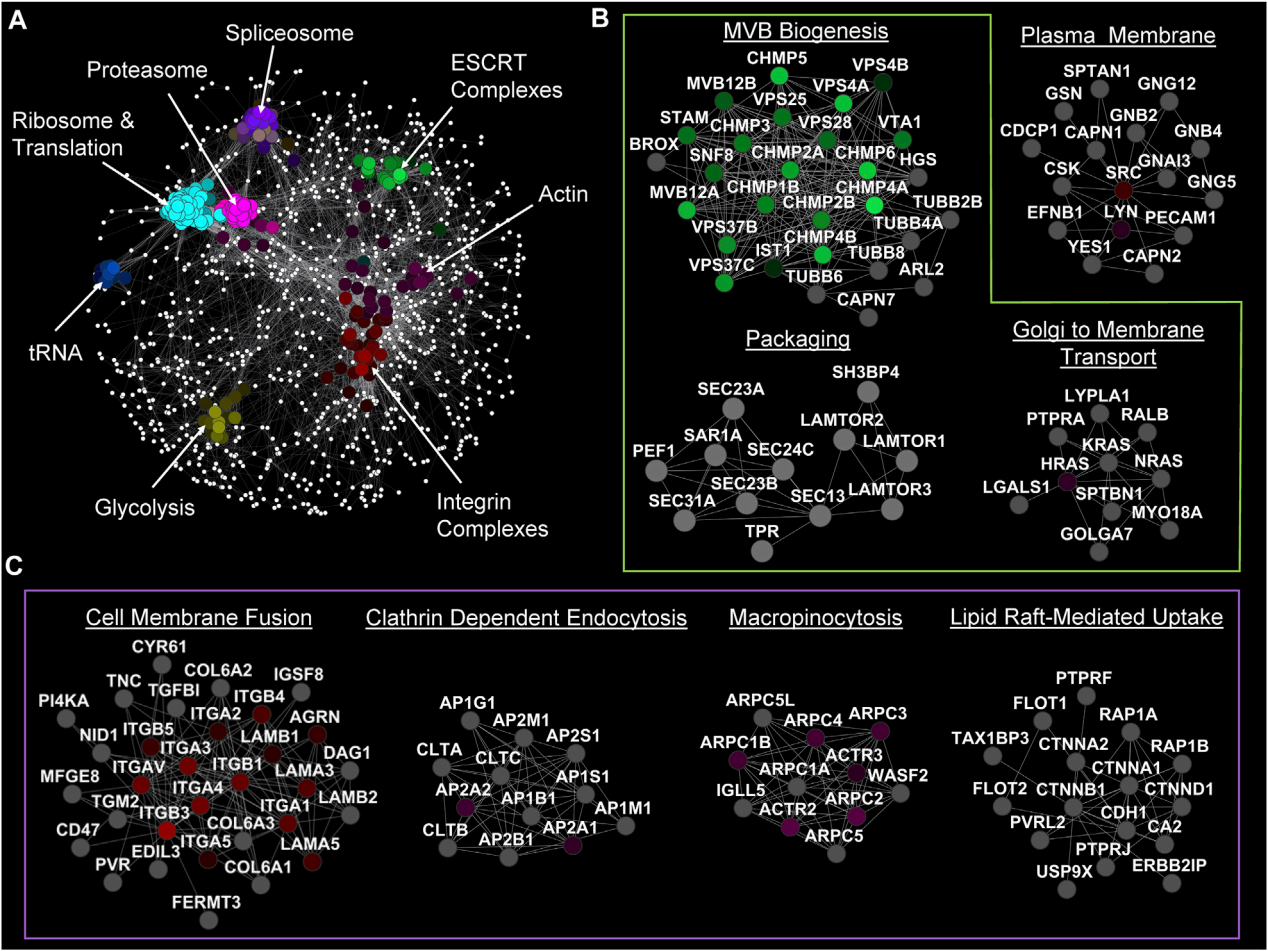
Network analysis of genes involved in EV biology. **(A)** SAFE analysis of genes that have appeared more than 73 times in articles (based on EVpedia). **(B)** Significant MCL sub-networks with crucial genes in EV biogenesis annotated. **(C)** Significant MCL sub-networks involved in EV uptake by different mechanisms.

To visualize and discover key players in EV biogenesis and uptake, we ran a comprehensive analysis of genes involved in these processes (Figure 2) from the EVpedia protein database (version 2018 April 30) (Kim et al., 2013; Kim et al., 2015a; Kim et al., 2015b), specifically focusing on human datasets. The frequency of each protein was calculated and fitted in a two-component mixture model of gamma and standard normal distributions, ranging from 1 to 410. The intersection of the gamma and standard distributions was established as a cut-off point at 73.52, which led to the selection of Uniport accessions that appeared 74 times or more. Out of 25,118 human proteins in the dataset, 1,670 satisfied this criterion. These proteins were further analyzed using the STRING (version 2.0.1) protein inquiry in Cytoscape (version 3.10.0) with a confidence cut-off of 0.9, which resulted in a significant network with 1,340 nodes. Spatial Analysis for Functional Enrichment (SAFE, version 1.0.0. beta7), which categorizes the network based on their Gene Ontology (GO) annotation (go_Hs_P_160509) by different colors, revealed many housekeeping genes that are crucial for both cell survival and EV biogenesis or uptake. Other enriched hits included genes involved in translation, tRNA aminoacylation, glycolysis, construction and maintenance of the cytoskeleton, and various activities within the spliceosome, ribosome, and proteasome (Figure 2A). Next, the MCL (Markov Cluster Algorithm) clustering method was used to find sub-networks, and each was named based on their functional annotation (Huang et al., 2009).

As expected, we identified ESCRT-0, -I, -II, and -III complexes that are vital for the biogenesis of MBVs and, therefore, for exosome biogenesis. Note that ESCRT-I and -III complexes are also involved in ectosome biogenesis. The genes identified within the “Packaging” cluster play crucial roles in encapsulating proteins into EVs, specifically facilitating their transport from the ER to the Golgi by COPII machinery (Bisnett et al., 2020). Furthermore (Figure 2B), it was identified that the RAS protein family involvement in Golgi to plasma membrane transportation affects EV biogenesis (Pantazopoulou et al., 2023).

This network analysis also detected the undeniable roles of clathrin subunits and clathrin assembly complexes in the formation of clathrin-coated vesicles and the delivery of EV cargo into recipient cells. Other than the expected players, there were a few other interesting complexes enriched in the sub-network analysis. For example, our analysis highlighted the secondary involvement of actin and integrin complexes, which are considered to be among the most important proteins in cells, in ectosome biogenesis and EV uptake. Integrins appeared as one of the main factors in the cell membrane fusion uptake pathway while macropinocytosis uptake pathway is dependent on the actin cytoskeleton. Similarly, genes in the “Lipid Raft-Mediated Uptake” cluster can first be viewed as a housekeeping cluster because of their role in cell adhesion. However, the presence of the flotillin family (FLOT1 and FLOT2) strongly supports their role in the internalization of EVs. Although we are not ruling out the potential role of lipid rafts in other forms of EV biogenesis and lipid redistribution (Figure 2C).

## 5 Discussion: role of EVs in disease diagnosis and prognosis, and therapeutic potential

EVs have promising potential as diagnostic and prognostic biomarkers in a spectrum of diseases, including chronic cardiovascular diseases and cancer. For chronic cardiovascular diseases, such as atherosclerosis, aortic stenosis, and aortic aneurysms, the diagnostic utility of EVs is linked to the detection of miRNAs such as miR-222, miR-143, etc., and proteins like HSP60, VCAM1, etc. (Martin-Ventura et al., 2022). Moreover, certain molecules like proteins, proteoglycans, and miRNAs in tumor-derived EVs have shown potential in the diagnosis of specific cancers (Melo et al., 2015; Giallombardo et al., 2016; Hu et al., 2022). Intriguingly, a subgroup analysis showed that the use of small EVs is more accurate as a biomarker in serum samples from nervous system cancer compared with other cancer types, hinting that small EVs in serum represent promising diagnostic tool development for nervous system cancers (Liu et al., 2021). Beyond their diagnostic potential, EVs can be utilized as promising therapeutic tools. One prospective avenue is their potential use for drug delivery. EVs loaded with therapeutic agents can be delivered to target cells. This approach offers several advantages over traditional drug delivery methods, like protecting the therapeutic agents from degradation and enabling precise targeting of specific cell types. This ensures increased efficacy and reduced side effects (Sun et al., 2023). Additionally, EVs isolated from existing stem cells have gained popularity in the field of regenerative medicine. For instance, EVs derived from mesenchymal stem cells (MSCs) show therapeutic potential in various diseases, including cardiovascular, liver, and kidney diseases, by promoting tissue repair and regeneration (Park et al., 2019). Notably, umbilical cord-derived MSCs have been utilized to treat COVID-19 patients (Alzahrani et al., 2020).

Considering cancer, EVs fine-tune the tumor microenvironment and may potentially play a pivotal role in cancer metastasis. Thus, the uptake of cancer-associated EVs could open new therapeutic areas. Previous research has identified phagocytosis as the most efficient mechanism for EV internalization from cancer and leukemic cells (Feng et al., 2010; Emam et al., 2018). This efficiency can be attributed to the diverse array of surface receptors displayed on phagocytic cells, allowing them to bind numerous ligands on EV surfaces, rendering phagocytes as highly suitable recipient cells (Wall et al., 2020). Consequently, targeting EVs to phagocytes can be advantageous for exploring novel treatment options. This strategy is not limited to oncology since immune cells significantly influence other diseases (Liu et al., 2017), such as Parkinson’s disease (Ren et al., 2023) and neuroinflammation (Verweij et al., 2021), wherein EV-mediated macrophage polarization or phenotype switching in microglia holds potential. To this end, significant attention has been devoted to developing camouflage strategies to prevent EV recognition by phagocytes. Introducing molecules such as CD47 or CD24 on tumor cell- or fibroblast-derived EVs shields them from phagocytosis (Kamerkar et al., 2017; Karnas et al., 2023). Other molecules like CD31, CD44, or β2-microglobulin are also being tested to enhance phagocytosis evasion further, thus extending the circulation half-life of EVs (Parada et al., 2021).

Lastly, EVs are being explored for cancer vaccine applications (Santos and Almeida, 2021). Specifically, dendritic cell-derived exosomes (DCexos) and tumor cell-derived exosomes (Texos) are being tested as cancer vaccine candidates. DCexos, which carry major histocompatibility complex (MHC) class I/II and co-stimulatory molecules, have so far demonstrated increased anti-tumor efficacy in pre-clinical models compared with dendritic cell vaccines; conversely, while Texos are attractive candidates for cancer vaccines, their inherent immune-suppressive functions pose challenges (Yao et al., 2021).
