# Supplementary figures and images for: Biogenesis and delivery of extracellular vesicles: harnessing the power of EVs for diagnostics and therapeutics
Source: Front Mol Biosci. 2024 Jan 3;10:1330400. doi: 10.3389/fmolb.2023.1330400 (PMC10791869; doi:10.3389/fmolb.2023.1330400)

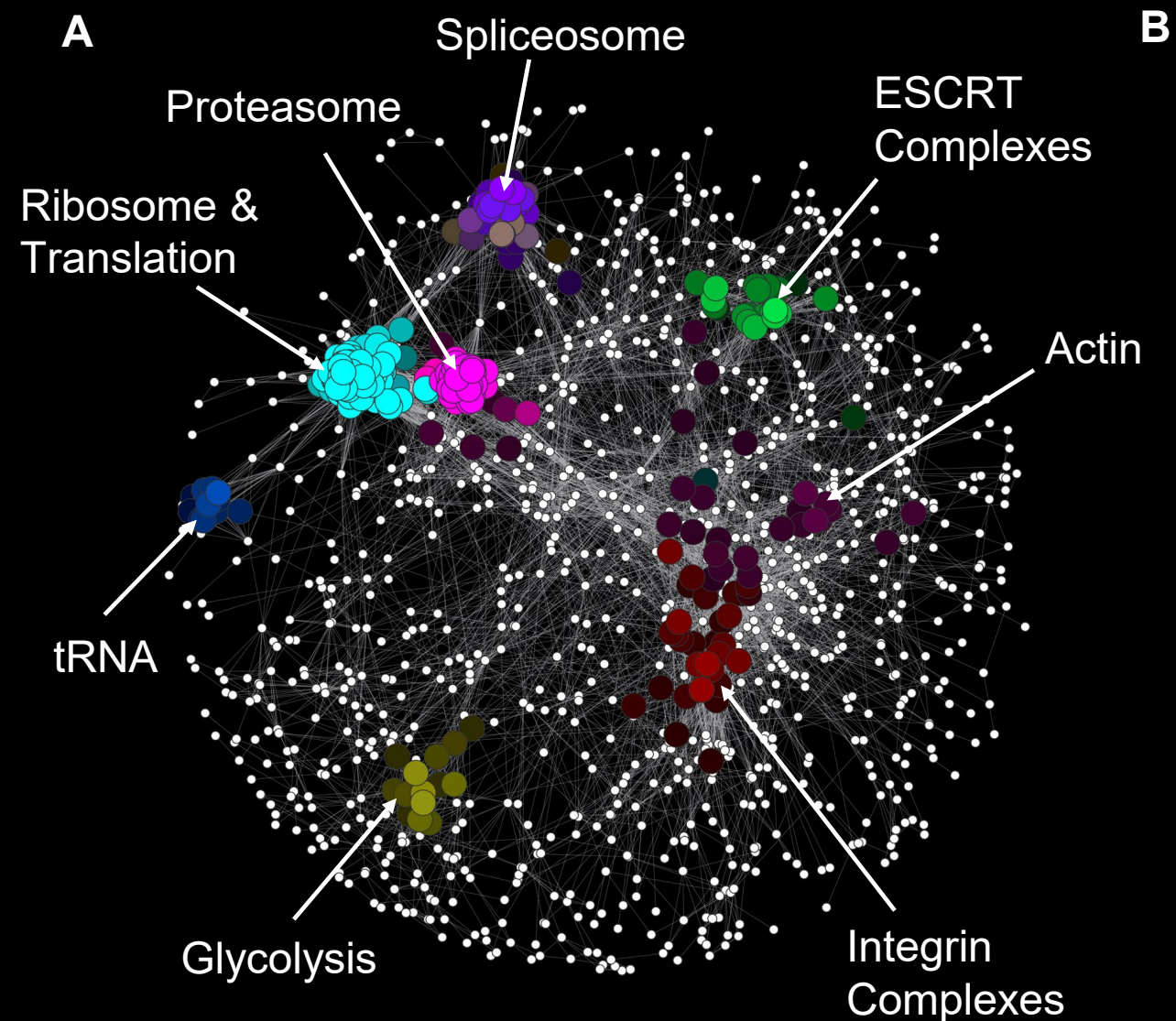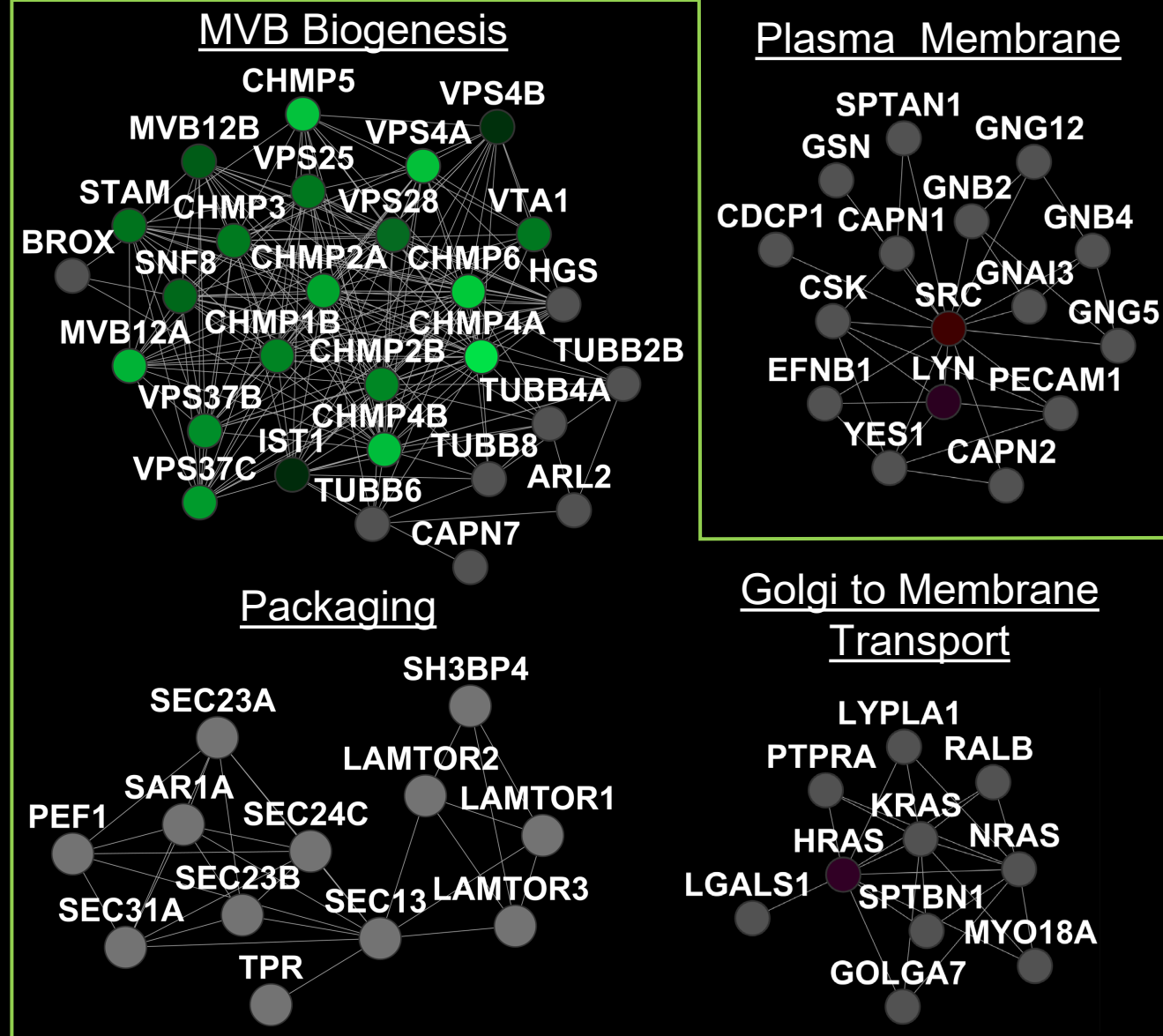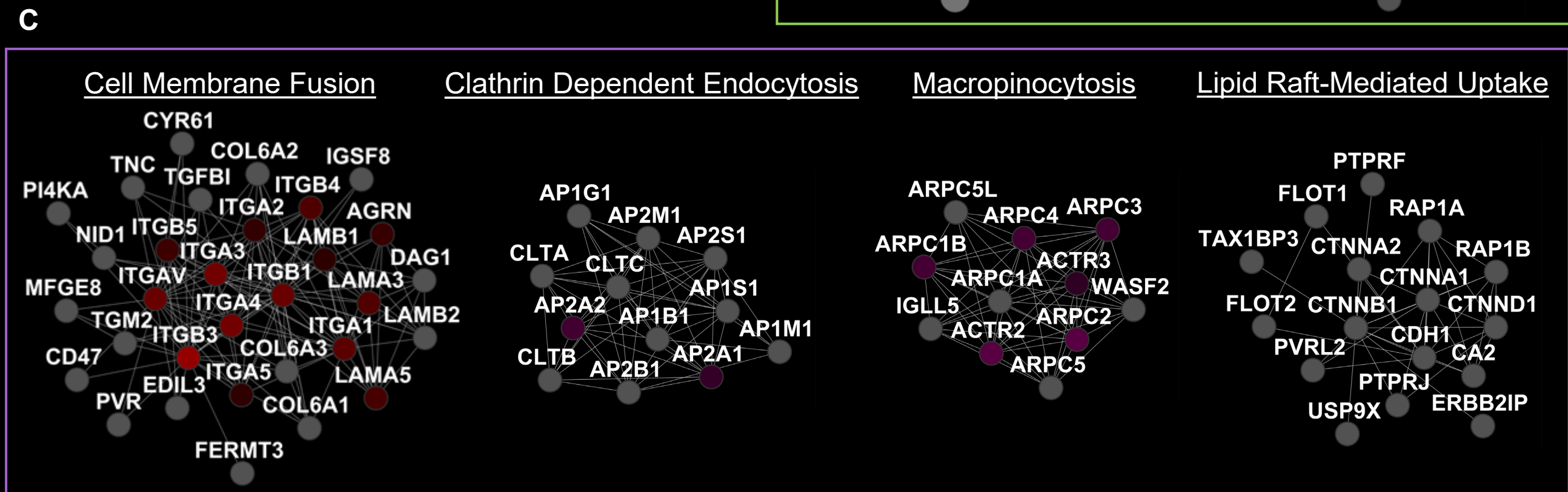

Supplement: Supplementary file 1 [file DataSheet2.PDF]

## A. EVs Biogenesis

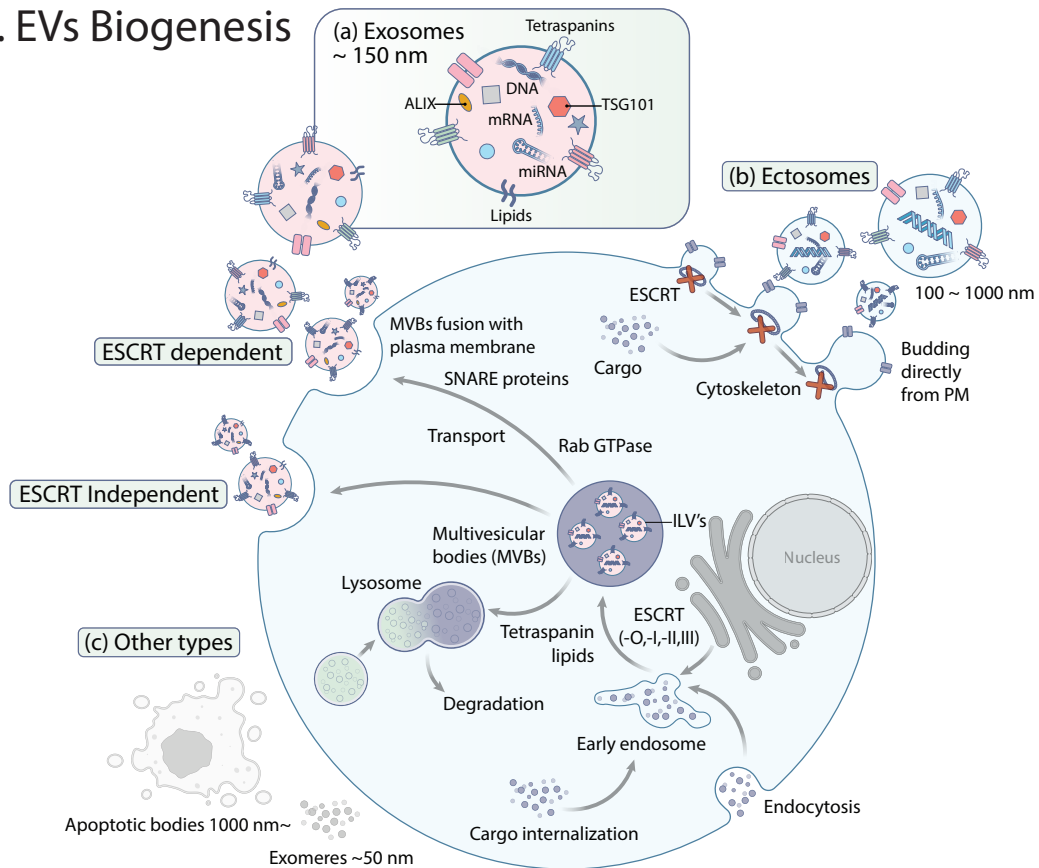

## B. EVs delivery

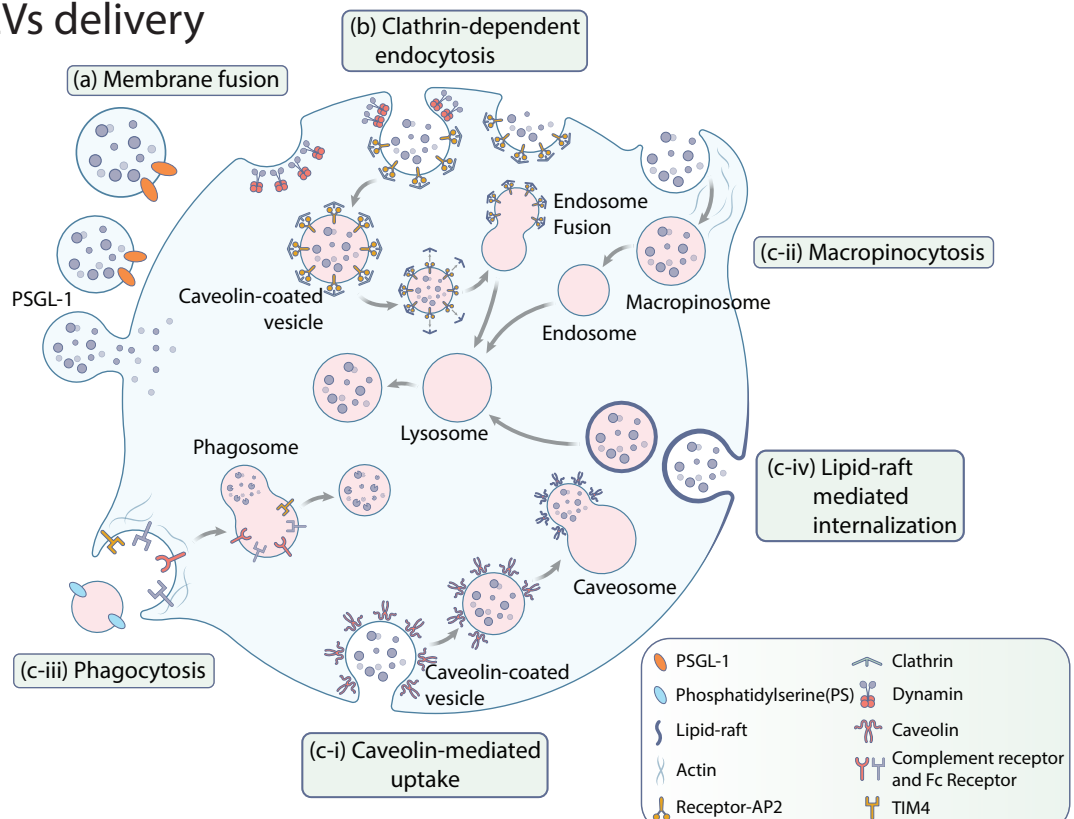

Supplement: Supplementary file 2 [file DataSheet1.PDF]
